# *Rickettsia sibirica* subsp. *mongolitimonae* Infection and Retinal Vasculitis

**DOI:** 10.3201/eid1404.070859

**Published:** 2008-04

**Authors:** Julie Caron, Jean-Marc Rolain, Frédéric Mura, Bernard Guillot, Didier Raoult, Didier Bessis

**Affiliations:** * Université Montpellier 1, Montpellier, France; † Université de la Méditerranée, Marseilles, France

**Keywords:** Rickettsia sibirica subsp. mongolitimonae, retinal vasculitis, pregnancy, letter

**To the Editor:** *Rickettsia sibirica* subsp. *mongolitimonae* is an intracellular bacterium that belongs to the species *R*. *sibirica* (*1*). To date, only 11 cases of infection with this bacterium have been reported (*2**–**6*). We report a case in a pregnant woman with ocular vasculitis.

A 20-year-old woman in the 10th week of her pregnancy was admitted in June 2005 to St. Eloi Hospital in Montpellier, France, with an 8-day history of fever, eschar, hemifacial edema, and headache. On examination the day of admission, she had a fever of 38.5°C, headache, and frontal eschar surrounded by an inflammatory halo. Painful retroauricular and cervical lymphadenopathies were noted. Results of a clinical examination were otherwise within normal limits. No tick bite was reported by the patient, although she had been walking a few days before in Camargue (southern France). Serologic results for *R*. *conorii*, *R*. *typhi*, *Brucella* spp., *Borrelia* spp., and *Coxiella burnetii* were negative.

One day after admission, she reported loss of vision (scotoma) in her right eye. She underwent a complete ophthalmic evaluation. Measurement of visual acuity and results of a slit-lamp examination were within normal limits, but a funduscopic examination showed a white retinal macular lesion that corresponded in a fluorescein angiograph to an area of retinal ischemia induced by vascular inflammation and subsequent occlusion (Figure). The following day, a rash with a few maculopapular elements developed, which involved only the palms of the hands and soles of the feet. Mediterranean spotted fever was suspected. Cyclines and fluoroquinolones were contraindicated because of her pregnancy, and the patient had a history of maculopapular rash after taking josamycin. She was treated with azithromycin, 500 mg/day for 10 days, under close surveillance. After 2 days of treatment, she was afebrile and the rash completely resolved. No obstetric complications occurred and she gave birth to a healthy boy at term. Two years later, the right scotoma remained unchanged.

**Figure:**
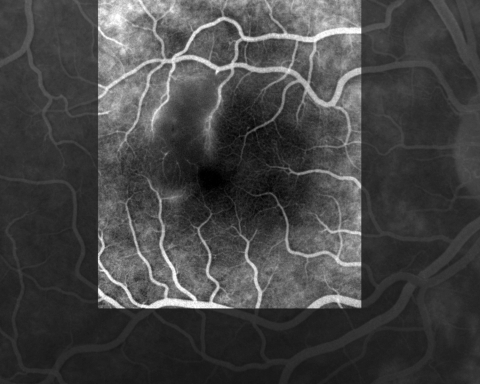
Fluorescein angiograph of the right eye of the patient showing retinal occlusive vasculitis with arteriolar leakage at late phase.

Serologic tests for rickettsiosis were performed with an acute-phase serum sample and a convalescent-phase serum sample (1 month after onset of symptoms). Samples were sent to the World Health Organization Collaborative Center in Marseille for rickettsial reference and research. Immunoglobulin (Ig) G and IgM titers were estimated by using a microimmunofluorescence assay; results were negative. Culture of a skin biopsy specimen from the eschar showed negative results.

DNA was extracted from eschar biopsy specimen and used as template in a PCR with primers complementary to portions of the coding sequences of the rickettsial outer membrane protein A and citrate synthase genes as described (*5*). Nucleotide sequences of the PCR products were determined. All sequences shared 100% similarity with *R*. *sibirica* subsp. *mongolotimonae* when compared with those in the GenBank database.

Infections caused by *R*. *sibirica* subsp. *mongolitimonae* have been reported as lymphangitis-associated rickettsiosis (*4*). Our case-patient had the clinical symptoms reported for this disease: fever, maculopapular rash, eschar, enlarged satellite lymph nodes, and lymphangitis. Seasonal occurrence of this disease in the spring is common and has been reported in 9 of 12 cases, including the case-patient reported here (*2**–**6*). A total of 75% of these *R*. *sibirica* subsp. *mongolitimonae* infections occurred in southern France; other cases have been recently reported in Greece (*5*), Portugal (*6*), and South Africa (*7*). However, the vector of *R*. *sibirica* subsp. *mongolitimonae* has not been identified (*7*). This rickettsia has been isolated from *Hyalomma asiaticum* ticks in Inner Mongolia, from *H*. *truncatum* in Niger (*8*), and from *H*. *anatolicum excavatum* in Greece (*5*). *Hyalomma* spp. ticks are suspected of being the vector and are widespread in Africa, southeastern Europe (including France), and Asia.

Rickettsiosis caused by *R*. *rickettsii* and *R*. *conorii* during pregnancy has been reported without risk for vertical transmission (*9*). First-line antimicrobial drugs used to treat rickettsial disease are cyclines and quinolones, but they are contraindicated during pregnancy. Chloramphenicol is an alternative drug for pregnant women but it is not available in France. Macrolides (azithromycin, clarithromycin, and josamycin) are effective against rickettsial disease and can be used safely during pregnancy.

No ocular complications were reported in the 11 previous cases of rickettsiosis caused by *R*. subsp. *mongolitimonae*. However, ocular lesions, including optic disk staining, white retinal lesions, retinal hemorrhages, multiple hypofluorescent choroidal dots, mild vitritis, and retinal vasculitis, have been described in patients with rickettsiosis caused by *R*. *conorii*, *R*. *rickettsii*, and *R*. *typhi* (*10*). Most of these posterior segment manifestations are usually asymptomatic in patients with acute Mediterranean spotted fever (*10*) and can be easily overlooked. Retinal vasculitis was reported in 45%–55% of the patients, but retinal artery occlusion secondary to vasculitis has been described in only 2 cases of infection with *R*. *conorii* and *R*. *rickettsii* (*10*) without details of clinical symptoms. Because ocular involvement could be asymptomatic and easily overlooked, an ophthalmic evaluation should be conducted when rickettsiosis is suspected.
